# Development of a Computational Policy Model for Comparing the Effect of Compensation Scheme Policies on Recovery After Workplace Injury

**DOI:** 10.1007/s10926-022-10035-w

**Published:** 2022-05-10

**Authors:** Jason Thompson, Camilo Cruz-Gambardella

**Affiliations:** Transport, Health and Urban Design Research Hub, The University of Melbourne, Melbourne, Australia

**Keywords:** Policy, Injury, Rehabilitation, Agent based model

## Abstract

*Introduction *The direct comparison of real-world workers’ compensation scheme management policies and their impact on aspects of scheme performance such as health and return to work outcomes, financial sustainability, and client experience metrics is made difficult through existing differences in scheme design that go beyond the factors of interest to the researcher or policymaker. Disentangling effects that are due purely to the result of policy and structural differences between schemes or jurisdictions to determine ‘what works’ can be difficult. *Method *We present a prototype policy exploration tool, ‘WorkSim’, built using an agent-based model and designed to enable workers’ compensation system managers to directly compare the effect of simulated policies on the performance of workers compensation systems constructed using agreed and transparent principles. *Results *The utility of the model is demonstrated through and case-study comparison of overall scheme performance metrics across 6 simple policy scenarios. *Discussion *Policy simulation models of the nature described can be useful tools for managers of workplace compensation and rehabilitation schemes for trialing policy and management options ahead of their real-world implementation.

**Supplementary Information:**

The online version contains supplementary material available at 10.1007/s10926-022-10035-w.

## Introduction

Australia has a rich history of creating world-leading, no-fault, compulsory third-party social insurance and injury compensation schemes [1, 2] whose role is to guard individuals against the worst consequences of injuries and disability generated through workplace accidents, road traffic crashes, and more recently, acquired and/or developmental disabilities [3]. Combined, these schemes (e.g., WorkCover, iCare, Transport Accident Commission, MAIC, ReturntoWorkSA & National Disability Insurance Scheme) pay out close to AUD $50 billion in costs of medical care, rehabilitation services and support per year, estimated to reach AUD $70 billion by 2024/25 [4, 5]. For context, this is equivalent to 3% of Australia’s gross domestic product and more than current expenditure on either Defence (~ AUD$50 billion) or Medicare (~ AUD$40 billion), which is Australia’s universal public health care scheme.

Australian injury and disability insurance schemes are therefore objectively expensive, but they are also vital to the operation of critical social systems that people rely on every day. Without access to universal, affordable insurance, people could not work or engage in trade, transport, or broader society without legitimate concern that ruinous financial, functional, or health-related consequences might result from unplanned road crashes, workplace accidents, or other misfortune. As previously witnessed [6, 7], when insurance schemes become unaffordable, unviable, dysfunctional or fail, significant societal unrest and widespread personal, community, and economic damage may follow. In particular, workplace injury compensation schemes provide an important social benefit; they act to prevent workplace injuries, assist injured workers to recover, and reduce the costs associated with injury for individual workers, organisations, business, and the broader community through effective and efficient management of a common pool insurance model. Maintaining the integrity and viability of these schemes is therefore critical for the health of individuals and society, alike.

For injury insurance and compensation schemes that hold dual roles of prevention (i.e., through safety regulation) and recovery, errors made on one side of the equation affect the organisation’s ability to effectively carry out functions on the other. On the prevention side, this can include failure to recognise or reduce workplace hazards through education, inspection and enforcement activities, which leads to elevated injury risks, rates and premiums [8, 9]. On the rehabilitation and recovery side they can include inefficient or adversarial injury rehabilitation system management processes, failure to provide access to timely treatment, or failure to find or support suitable roles for injured workers that enable a graded return-to-work [10–15].

Today, many of the issues described above are affecting Australian workplace injury and social insurance schemes, placing them under various levels of threat [16]. For example, as well as experiencing significant cost blow-outs generating tens of billions of dollars in financial liabilities [4, 17], efforts to limit expenditure in some workers’ compensation schemes is failing [18], and in others is leading to well-documented pursuit of legislation, performance incentives, and aggressive management practices that then produce damaging functional, financial, and psychological outcomes for injured clients and people with disabilities and their families that are at odds with schemes’ intended purposes [11, 19–21]. Given the serious potential society-wide consequences of workplace injury prevention and rehabilitation system-generated mistakes and mismanagement, a consistent focus on improvement of workplace injury prevention and management systems so they can (1) improve the health of injured people, (2) meet their non-medical needs, and do so in a (3) financially fair and reasonable manner—as per recognised hallmarks of high-performance health systems [22, 23]—is warranted.

Challenges remain, however, in exactly how to achieve and maintain levels of high performance over time, particularly in a dynamic economic, cultural, technological, and legal environment. However, instead of relying on natural or in-situ experimental methods that may take months or years to play out, or cross-jurisdictional comparisons where differences between schemes may be too great for fair comparison [24], it is possible that dynamic policy modeling simulators—devised using agent-based and other computational modeling frameworks—may help in aiding better decision-making [25–28].

This project developed a policy simulator for workplace safety and compensation system management, designed around the function of an Australian state-based workers’ compensation scheme (The Scheme). The goal of the project was to create a policy simulator using an agent-based model that enabled scheme managers to compare the effect of various combinations of health, safety, and scheme policy settings. The model was designed to assist understanding of dynamic relationships between system settings, actors and outcomes, and how these can be combined to optimise scheme performance. Further, the model was designed to act as a first iteration that can be built upon, describing and recording a virtual history of scheme design and management policy, and demonstrating the utility of this approach as a learning and decision-support tool.

## Method

### Agent-Based Modelling (ABM) and Computational Social Science

Computational social science is the discipline of representing communities, societies, and social phenomena through the generation of tangible, observable, but computer-generated ‘artificial’ societies, often using agent-based models (ABMs). By representing critical structures, relationships, and dynamic interactions between independent computational agents within artificial societies, circumstances and phenomena representing the antecedents and outcomes of realistic crises befalling a system or society (e.g., a pandemic or collapsing social insurance scheme) can be generated [29]. Similarly, if realistic crises within artificial societies and systems can be generated, so too can synthetic policies that either prevent those same events from arising or provide solutions to emerging or unfolding crises. Modeled policies can also be generated that optimise the performance of systems across potentially competing domains. Prior efforts that have used computational approaches include system dynamics models [30]. However, while often comprehensive and transparent, system dynamics models can struggle to appreciate levels of heterogeneity among injured populations that are critical to understanding the behaviour of systems. This is where agent-based modeling—which takes an individual, bottom-up approach—is of greatest value. Computational social science is a nascent but active field that demonstrates great potential in predicting, understanding and solving many problems facing contemporary society within complex socio-technical systems [31]. It has gained particular attention during the COVID-19 pandemic through its contribution to understanding patterns of disease spread and its ability to generate policy scenario models that surpass traditional epidemiological methods’ capacity to reflect the effect of dynamic Government strategy [32–34].

### Model Design and Build

The model was built through collective, iterative understanding from staff, management, researchers, and available research evidence of The Scheme’s operation. The process of model development went through 10 stages, described, below.

### Stages of Model Development

**Table:** 

Stage	Description
1. Ethics application	An ethics application was submitted to the University of Melbourne. The project was approved under Ethics application 1852544.1
2. Problem formulation and identification	An initial 2-h workshop was conducted with 3 Scheme representatives, defining the scope and aims of the model. It was determined that the model should focus on the high-level pathway of injured clients from initial claim acceptance through to recovery, with a focus on policies relating to the introduction or application of Occupational Rehabilitation services
3. System identification and decomposition	At the initial workshop, participants identified important actors, structures, pathways and influences that could be included in a high-level model. Participants were also presented with a simplified example of agent and model behaviour to assist understanding of how agents might interact and move within the model. This began a process of building both a causal loop diagram with the scheme representatives that featured shared understanding of the incentives and direction of effect of high-level concepts and factors in the scheme, as well as a state-chart which would describe the ‘position’ in the system that an individual worker might hold at any point in time
4. Concept formalisation	Over the course of seven sessions, interviews and teleconferences were held with scheme representatives to define relationships, behaviours and interactions of the model actors using the causal loop diagram and the state charts (see Supplementary Appendix A and B). At each meeting, participants returned to the causal loop diagrams and state charts and iterated them until either consensus was reached or agreed deviations or changes to the diagrams were made. Between meetings, the research team attempted to operationalise these charts in basic computational models to check for logic and validity errors. Where identified, they were addressed at the subsequent meetings with scheme representatives. This process continued until an agreed point was reached that satisfied the scheme representatives and researchers that all factors of greatest importance had been included in the model and none excluded for the purposes of the project and consistent with the (limited) resources allocated to it
5. Model formalisation	The model formalisation phase reflected the combined state charts and relationship chart as far as possible within the time and resource constraints of the project. The model ‘story’ was then generated from the static state charts and causal loop diagram and transcribed into pseudo-code
6. Software implementation	The model was coded into the NetLogo agent-based modelling platform. A parallel model was also attempted in the Godot game engine platform. The code and model can be accessed via GitHub https://github.com/JTHooker/WS_ABM by request. The annotated code and interface is included in Supplementary Appendix D
7. Model verification	The model was iterated and run under various baseline and experimental conditions to determine whether the actors and relationships were operating as expected. In its current version, the model is operating satisfactorily with no obvious bugs. All settings and levers are operating as expected and the model is stable. The model is not (and can theoretically never) be regarded as complete. The model should only ever be regarded as ‘sufficient’ for understanding or addressing given problems that are reasonably within the scope of its design
8. Experimentation	Depending on definitions, the model currently contains around 25 policy and claims management levers and literally trillions of potential policy combinations with an equally high number of potential outcomes experienced over time. The ability to test all combinations is obviously beyond the scope of the current project, however, a combination of two policies were more formally tested. 1) The promotion of return to work at work or not (three settings), and 2) providing support for return to work at work through additional occupational rehabilitation provider assistance. Further available policy settings able to be manipulated include claims acceptance thresholds, changes to the duration of eligible claims prior to the termination of benefits, dispute resolution rates, funds spent on advertising for safety promotion and encouraging recovery at work, accuracy of medical diagnoses, workers’ expectations of waiting times for claims lodgement and assessment, GP and Emergency care referral rates, and claims acceptance thresholds. Outcomes of the system can be measured at the level of individual claimants or as aggregate treatment and wage costs, advertising spend, duration of claim, duration of decision-times, RTW outcomes, worker satisfaction and trust in the system, volumes of claimants in various RTW and other states, and overall treatment, wage replacement, and system costs, among others
9. Data analysis	Data produced by the model was analysed to identify differences in system performance across the combination of policy-settings described in (8), above
10. Model validation	Model validation is ongoing, acknowledging that within agent-based models, validation is not easily defined [35, 36]. In its present state, it may be considered mechanistically valid given that it take a form that is agreed upon by scheme managers. To the best of our knowledge, it therefore validly and realistically explains the dynamics at work within the scheme. The outputs it produces (e.g., actual $ figures and estimates), however, have not been calibrated and should not be used for forecasting raw numbers

### Model Narrative

**Fig. 1:**
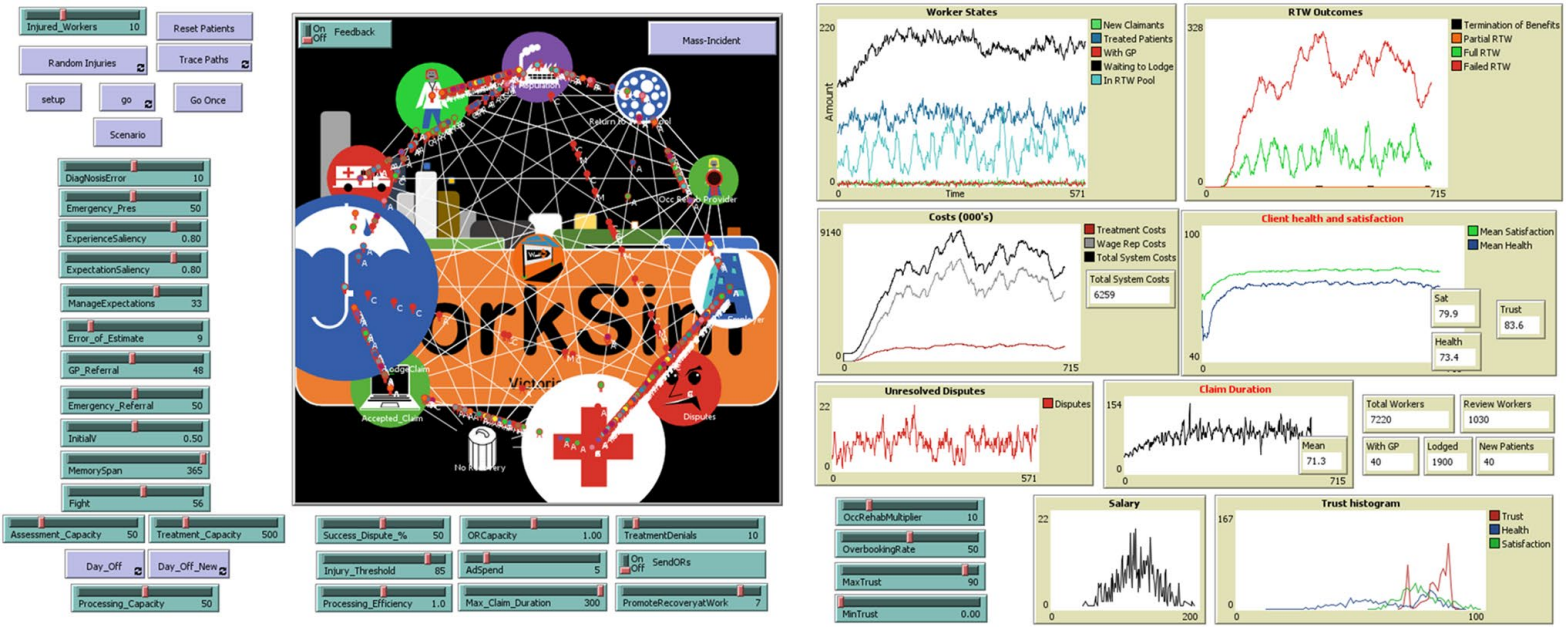
Model interface, showing all model features, policy levers, inputs, charts and monitors

In this model, the operator is referred to as ‘The Observer’. In the context of model use, the Observer would represent a person who is using the model (e.g., a staff member, manager or management group) to test policies. The model interface is shown in Fig. 1.

Injured workers were represented in the model as small, multicolored icons. Thousands of injured workers could be contained within the model at any time.

Injured workers entered the model from employers at a rate of 10 clients per time-step, emanating from the ‘general population’ depicted by a factory icon at the top of the model interface. In models of this kind, time is recorded as a unit but is not strictly calibrated to any real-world time-period (e.g., a day, week, or year). The number of incoming injured workers could be set to vary over time randomly up or down 1 unit every 50 time-steps, reflecting variation in the rate of injury among workers between periods.

Injured workers had a range of qualities upon entrance that were unique to them as individuals. In some cases, these were scaled to represent qualities out of a maximum of 100 points (the use of ‘100’ points is arbitrary but acts as a standard 100-sided die which can rolled for any individual at any time and compared against another number in the model that may act as a threshold above or under which actions might be taken). For example, they entered with levels of:

- overall health out of 100 points;
- satisfaction with the system out of 100 points;
- trust in the system out of 100 points; and
- an appetite for fighting unfavourable decisions out of 100 points.

Other qualities that injured workers had included:

- a level of responsiveness to treatment that enhanced or discounted the effectiveness of any treatments applied to them by a multiplier (between − 1.0 and + 1.0);
- a memory and memory span that could record past events (of up to 300 time-steps);
- a set of expectations relating to timeliness of service understood as time-steps (e.g., an injured worker may expect to have their claim processed within 35 time-steps);
- a type of work injury claim suffered (acute physical, chronic physical, or mental health);
- a salary (mean of $55k per year and a positively-skewed distribution with a standard deviation of $10k); and
- a duration of time that their injury claim had been active.

All workers who entered the system had an injury, defined by a level of health that was below 100 total points. After injury, there was a 50% likelihood that injured workers first reported to a general practitioner (GP), and a 50% likelihood that they reported to an emergency ward or hospital. These proportions could be manipulated by the model user. Costs associated with either a GP visit (average of $100) or an emergency visit ($1000) were recorded against the individual worker. These costs were not recorded against the system unless the worker’s injury claim was later accepted (see below).

For injured workers who were treated by a GP, the next stage of referral could go in two directions. GPs assessed the incoming injured worker, and if the worker’s health was assessed as lower than the claim threshold set by the system for a legitimate workplace injury claim, they were sent to the claim lodgment stage. Otherwise, they were sent back to the Workplace. However, GPs did not always make a correct diagnosis, and occasionally sent workers back to work when they were unfit to return. Upon arrival at the workplace, if the worker had not achieved a health status above the claim threshold, they were then referred back to the claim lodgment stage and lost a degree of trust in the system. If they had achieved a health status above threshold (workers’ health varied at each time step but was likely to incrementally improve each time-step in line with a small waitlist effect), they moved back toward the general working population and were removed from the compensation system.

When injured workers reached the claim lodgment stage (blue, umbrella icon), they were held there for the duration it took their claim to be assessed. This time delay (if any) was dependent upon the volume of claims that were being concurrently processed and the capacity of the administrative function of the scheme to process any new incoming claims.

The likelihood that an injury claim would be accepted was based on an interaction between an injured worker’s injury type (acute physical, chronic physical, or mental health) and the claim acceptance threshold of the agent. Acutely injured workers were likely to have their claim accepted at a rate of 90%, while workers with chronic injuries and mental health claims were likely to have their claim accepted at base rates of 60% and 40%, respectively. These acceptance rates and the processing capacity of agents could be adjusted by the observer in-line with desired policies or observed current practice. If injured workers’ claims were not accepted, they lost trust in the system over time, raising the likelihood that they would later become adversarial and decide to dispute future unfavourable decisions. If workers decided to contest a denied injury claim, they moved toward the disputes area where they could have the decision reviewed. If not, they gave up and were removed from the system. Workers in dispute processes (those moving to dispute areas and existing within dispute areas) lost further trust and satisfaction in the system over the period they were in these states.

Workers in the dispute stage that had come from the claim lodgment stage could move in two directions. If their application to have their injury claim was successful, they moved back to the claim lodgment stage and tried again to have their claim accepted—this process could potentially repeat many times. If their claim was ultimately unsuccessful, they moved toward the general population and out of the Scheme system.

Once an injured worker’s claim was officially accepted, the date of acceptance was recorded and calculation of the duration of their claim began. Workers with accepted injury claims were then able to seek medical treatment if capacity existed in the treatment area. If there was no spare capacity, the injured worker waited in the accepted area until capacity in the treatment area became available. Treatment capacity could also be manipulated by the observer. Generally, treatment areas contained the highest number of injured workers at any time, reflecting the population of on-going injured worker claims in receipt of treatment. A further setting was also implemented that modelled short-term variation in assessment and treatment capacity due to intermittent staff shortages or processing bottlenecks.

Injured workers received treatment in the treatment centre, which improved their health by an average of 5% of the difference between their current health status and their maximum possible health status (100 units) per time-step, modified by the injured worker’s individual responsiveness to treatment score (e.g., 1.0 health units × 0.8 responsiveness units for injured worker x) plus or minus random 1 unit each time step. This meant that injured workers’ health improved faster in the early stages of the claim than later as they neared full recovery. For every treatment service received, an average cost of $100 units (sd $20 units) was recorded against the worker and added to aggregate medical treatment costs for the system. Additionally, wage replacement costs of 80% of the injured worker’s salary were recorded against the worker and system.

Occasionally, treatment would be denied to the injured worker at a rate that could be manipulated by the observer. When treatment was denied, the health of the injured worker did not improve (except by random change) and the level of trust that the worker had in the system decreased. Other observable (but indirect) effects of treatment denial were a rise in the number of Disputes and a reduction in the mean health of injured workers. Again, however, the injured worker could dispute this denial of treatment service. The likelihood that workers disputed treatment denials was a function of their trust in the system and willingness to fight decisions. Lower trust and more willingness to fight increased the likelihood of dispute. If successful, they moved directly back into the treatment system. If not, their trust in the system was diminished alongside their satisfaction and they moved back into the treatment system through the pathway of Claim Acceptance.

#### Returning to Work

Eventually, after receiving treatment and having had time off after their injury, most clients attempted to return to work. The conditions under which they did this were based on them reaching a level of health that was equal to or above the eligible claim threshold set by the system. For example, if the eligible claim threshold was 95 health points, at the time that an individual worker reached this level of health, they could move to the RTW pool and re-enter the workforce in full capacity (i.e., full RTW).

So, while movement from treatment to RTW was one possible pathway to RTW, the observer could also encourage injured workers to RTW through their employer prior to them reaching full health. Using this method, the observer could send injured workers to their employer at levels of health below the claim threshold (e.g., prior to full recovery). However, if the employer had a level of readiness to accept the worker (a quality of employers that could be added to make up the difference in capacity between the worker’s current health shortfall and the claim threshold), then that worker could make a partial RTW. If the employer was not ‘ready’ to accept the injured worker, they returned once again to the treatment stage and this was recorded as a ‘failed RTW’.

The encouragement of injured workers to return to their employer in the earlier stages of their recovery resulted in a high proportion of failed RTWs. Two strategies were then employed to counteract this. The first strategy involved activating a switch in the model that sent occupational rehabilitation support to employers to assist the employer to make up the shortfall in health status between the Worker and their capacity to work. The capacity of Occupational Rehabilitation support was able to be manipulated by the observer. Occupational rehabilitation support costs the system $10,000 per worker but resulted in a reduction of failed RTWs and an increase in partial RTWs, which then reduced wage replacement costs by an amount equal to the proportion of health the worker had achieved (i.e., if the injured worker was 75% fit, then wage replacement costs were reduced to 25% × 0.8 of salary).

The second strategy for increasing readiness of employers to accept injured workers back to work before they had achieved full health was to increase advertising spend in the system. Advertising spend had the effect of increasing readiness on the part of employers, encouraging them to expect Injured workers to return and to consider alterative or modified duties they could perform.

The final stage of the model involved workers moving from the RTW pool back to the general population. There were no special circumstances under which injured workers would not be able to return the population at this stage, except in the event that the duration of their claim surpassed the maximum time-limit, which was also able to be manipulated by the observer.

There are a number of other features of the model that are not described here but are best understood through interaction in a live environment. These include advertising spend on prevention activities, the activation of mass-industrial workplace incidents, the spread of mistrust and dissatisfaction between Injured Workers who share stories with one another, changes to termination of benefits criteria, and expectation management. Additionally, the monitors and charts associated with the model are important to understand. The GUI interface (Fig. 1) demonstrates the range of performance tracking features associated with the model. These charts are all modifiable and can be altered to suit the needs of the Observer.

## Results

Figures 2 to 9 in Supplementary Appendix C show just 9 example charts produced using data generated from the ‘BehaviourSpace’ feature of the model; a generic feature available within NetLogo that enables experimental conditions to be set up, run, and compared. In this example series of experiments, we manipulated just two features described above. Firstly, the encouragement (or neutral, or otherwise) for Injured Workers to complete their return to work at their Employer prior to achieving full health, and secondly, the provision (or otherwise) of occupational rehabilitation support to employers and injured workers who attempted to RTW through their employer. This produced a six-condition matrix between which eight performance variables were compared over a period of 2000 time-steps and results selected at the final time-step.

1. The mean total number of injured workers in the system
2. Mean satisfaction of injured workers with the system
3. Mean trust of injured workers in the system
4. Mean medical treatment costs
5. Mean total system costs
6. Mean wage replacement costs
7. Mean claim duration of workers at exit, and
8. Mean health of workers

The charts below should be interpreted as means for comparison between experimental conditions in relation to the direction of effects, only. At this stage (and prior to real-world calibration), we do not consider that the magnitude of difference between conditions is easily interpretable. However they do point to the interesting and complex nature of the modelling exercise and the fact that results produced by the model are not always immediately intuitive. We will take each of the results in turn.

The count of total injured workers in the system at the end of the experimental period provides insight into the efficiency of the system. A high number of injured workers in the system indicates that bottlenecks may exist in terms of processing delays or otherwise that is hindering people’s progress toward returning to work. The figures from our 6-condition matrix indicate that the most efficient policy settings for the system was one that promoted recovery in a treatment setting, rather than attempting to return through the employer (see Supplementary Appendix C, Fig. S1). Perhaps surprisingly, the poorest performing policy settings were associated with active encouragement of RTW at the employer while also providing additional occupational rehabilitation support.

Trends in the count of workers, above, are broadly reflected in the mean claim durations at exit for injured workers (see Supplementary Appendix C, Fig. S2). Again, settings that promoted recovery outside the employer produced the fastest mean RTW rates. The explanation for this may be that returning to work through the employer introduces potential additional processes and complexity into the recovery pathway that takes time away from a focus on improving health in the model.

These trends are again reinforced in the analysis of mean numbers of injured workers moving through the RTW pool at each time-step. Policy settings that encouraged recovery outside the employer were able to achieve the highest through-put of workers among the experimental conditions and assumptions in the model (see Supplementary Appendix C, Fig. S3).

Theoretically, longer claim durations should result in higher treatment and wage replacement costs as injured workers access more treatment services and have a longer period of wage support. Results support this contention within the model as treatment costs, wage replacement costs, and total system costs are elevated under conditions where recovery at work is promoted and this translates into higher claim durations (see Supplementary Appendix C, Figs. S4–S7). It is interesting to observe that higher wage replacement costs are still observed under conditions where the proportion of wage replacement is reduced during recovery (i.e., the promotion of recovery at work). However, it should be noted (again) that ***results should be taken as evidence of model verification*** more-so than true experimentation. If results were at odds with those above, it would highlight internal issues with the model coding and structure that need to be resolved. Assumptions and unit costs within the model will require calibration prior to use for policy decision-support.

So far, if we go back to our original premise that high performance health systems are reflective of those that are characterised by improving the health of injured people, meeting their non-medical needs, and doing so in a financially fair and reasonable manner, we could be tempted to say that policies promoting recovery away from work and not providing occupational rehabilitation support are those that are at least achieving equitable health and RTW outcomes for injured workers in the most financially fair and reasonable manner. However, are there trade-offs we are not considering in relation to levels of health and Injured Workers’ experiences of interacting with the system? Specifically, do these policy settings also meet Inured Workers’ non-medical needs?

Supplementary Appendix C, Fig. S8 shows the mean levels of satisfaction injured workers have with the system, while Fig. S9 shows the mean level of trust that workers have in the system whether they have had their claim accepted (blue) or rejected (orange). Minimal differences are observed across groups when the entire population is sampled, which is reflective or evidence from the real-world. However, delving into differences between groups that experience different processes (e.g., having a claim accepted or rejected), provides greater insight into the potential issues caused by various system processes and policies.

## Discussion

This paper describes the development of a policy simulator for workplace safety and compensation system management, designed around a no-fault workers’ compensation scheme that enables comparison of various potentially enacted policies.

The goal was to create a pilot version of an iterative policy simulation model that enables managers of workers’ compensation schemes to directly compare the effect of various health, safety, and policy settings prior to such comparison in the real world. The model was designed to assist understanding of dynamic relationships between system settings, actors, and outcomes, and how these can be combined to optimise scheme performance. Further, the model was designed to act as a first iteration that can be built upon in subsequent versions, with the potential to describe and record a virtual history of system design and management policy.

A significant advantage of using artificial societies to test policies and well as generate and solve crises that may strike workplace injury insurance schemes is that any failures experienced in the computational representations are divorced from the real-world. This enables ‘thought experiments’ or detailed ‘war-gaming’ to occur in a safe, off-line environment that can assist schemes prepare prevention strategies on the chance that these crises strike in the real world. For example, in an artificial society, a series of events or example scenarios could play out whereby a natural disaster or other calamity strikes leading to: (i) thousands of unexpected compensable deaths and injuries among workers, (ii) tens of thousands of insurance claims could then result, (iii) the costs of managing schemes spiral upward, (iv) insurance premiums increase dramatically for policy-holders, (v) thousands of businesses find their premiums unaffordable, resulting in (vi) business closures or re-location to other jurisdictions with lower rates, which, (vii) reduces contributions to the total insurance funding pool, which, (viii) results in inflated premiums for remaining policyholders and exit from the scheme.

In the real-world, such insurance scheme ‘death spirals’ [37] are potentially catastrophic. But in a computational world as described in this project—they are simply instructive and can provide lessons for shared and rapid improvements in scheme design. By viewing the outcomes of thousands of model runs, or by engaging directly with individual model runs under dynamic workshop conditions, computational policy models become tools that enable scheme managers, regulators and administrators to safely fail, learn, observe, and respond to the behaviour of realistic systems and schemes, as well as likely outcomes of proposed designs or policy changes, prior to taking action in the real-world [26, 27].

Beyond modeling the effect their decisions might have on the external world, another advantage of modeling artificial societies for injury and disability insurance scheme managers is to better understand impact that an uncertain and dynamic external world might have on them. For example, in 2021, a High-Court decision in the state of South Australia set legal precedent for additional compensation to be paid to claimants who have suffered secondary injury resulting from an original trauma. South Australian political and business leaders [8, 38] responded by suggesting the court’s ruling would undermine the viability of that state’s workplace injury compensation scheme (ReturnToWorkSA), creating a AUD $100m increase in claims costs per year, AUD $1.1b in forward liabilities and impose an additional AUD $300m hit to their operating position which had already recorded a loss of AUD $785 m in 2020/21 [17]. Added to this were concerns that consequent insurance premium hikes could force South Australian businesses to close or relocate interstate, resulting in the loss of 20,000 jobs over 5 years [8]. But whether such dire predictions are reasonable is difficult to judge.

However, if an open, transparent, agreed, and explicit [36] computational representation of ReturnToWorkSA—or any alternative national or international scheme—was available, a more objective assessment of the impact of the Court’s ruling both on the operation and affordability of ReturnToWorkSA, as well as effects on injured workers, business and employment conditions could be made. Further, an integrated model could assist all stakeholders to agree on how best to respond and maintain the integrity of the scheme by delivering on its individual and social performance goals [22].

Such a modeling framework could also directly assist stewards of workers’ compensation schemes in the Australian states of New South Wales (NSW) and Victoria that have been identified by the NSW Auditor General, State Insurance Regulatory Authority (SIRA) [11], and Victorian Ombudsman [19, 20], respectively, as falling far short of expected performance standards.

For example, in May 2021, NSW’s state-owned workers’ compensation insurance agency ‘iCare’ tabled financial reports for 2019–20 [18] estimating operating losses of AUD $2.7billion. These figures are set against a backdrop of acknowledged underperformance against return-to-work rates and recovery targets for injured workers, increasing claim costs, compensation underpayments to 53,000 workers, poor customer service, under-skilling and significant staff turnover among claims agents [10, 11], allegations of corruption [21], AUD $5billion losses in the prior 3 year-period [39], and the need for a AUD $4billion financial rescue package from NSW Treasury (i.e., taxpayers). Despite this, or perhaps because of it, workers’ compensation and 3rd-party injury insurance premiums in NSW remain at 33-year lows.

In Victoria, similar challenges exist where that state’s ‘WorkSafe’ scheme contends with a AUD $539million operating loss in 2019/20, a rising proportion of primary and secondary mental health injury claims, and recommendations from scathing 2016 and 2019 Ombudsman’s reports into scheme operations [40]. These reports [19, 20] detail how scheme design and incentives for reducing claims costs and improving return-to-work rates also incentivised aggressive claims management processes, unfair and unjustified claims decisions, and created adverse financial, physical and psychological outcomes for many injured workers. A transparent, explicit computational representation of both NSW and Victorian schemes could assist all parties to better understand scheme designs and mechanisms driving perverse incentives and producing poor performance as well as policy levers able to reverse these trends.

Limitations and challenges this project encountered primarily relate to issue of model validation and those associated with the software development and implementation.

Validation of models in computational social science—and particularly using agent-based models—is a scientifically ‘hot topic’ with many pages of formal and informal discussion and argument devoted to it (e.g., [36, 41, 42]). Validation is likely to be especially controversial when models are used in high-profile decision-making, whether that be in infectious disease [33, 34] or workers’ compensation system management. Broadly, however, we consider that validation arguments can be divided into 2 camps: (1) validation of mechanisms, and (2), validation of outputs. Validation of mechanisms can also be split into two groups: (1) expert validation of mechanisms, and (2), empirical validation of mechanisms. In this project, we have validated the model to the extent that it is transparently described in conceptual diagrams and code and that these representations are consistent with expert review provided by scheme managers. Additionally, the outputs generated by the model are consistent with the expectations of these same experts. We stop short of claiming, however, that the model is valid in terms of the outputs it generates as we have made no attempt to calibrate the model to real-world data. For further high-level discussion of these issues in agent-based modeling and computational modeling in general, we refer the reader to Epstein [36], Hassan et al. [35], Edmonds and ní Aodha [43], and Calder et al. [25].

Limitations from a software perspective were that, in addition to building the model in Netlogo, our desire was to also create a functional parallel model in a general purpose programming language. While we have achieved the goals of the Netlogo model, we continue to develop the general purpose or game-engine programming language version. However, this is challenging. General purpose languages (e.g., Godot, Python, Julia, etc.) are flexible but not purpose-built agent-based modelling packages. Consequently, primitive functions that are readily available in the NetLogo agent-based modeling language need to be built often from scratch in languages that have more accessible user interfaces for end-users or are more familiar to general programmers. This is an especially time-consuming task whose duration is difficult to estimate in advance. However, our research team is committed to developing this capability because we see perhaps the greatest challenge in this area as not building the models, themselves, but producing them in a format that is fast, engaging, and convincing for policy-makers [44, 45]. Even in this current model version, we have created an interface that improves upon the look and feel of previous models of similar kinds used in other jurisdictions and injury insurance contexts [27] by creating customized iconography and animation for agents within the model where their function is more explicit.

The next stage for the development of this model is further testing and calibration of it with staff and managers from real-world schemes so that it can be developed into a strategic decision-support tool. This has already occurred informally within workshop conditions with managers of schemes across Australia but is yet to be formally evaluated. Further work will involve its iteration and expansion to tackle agents and employer behaviour issues of contracted claims management more deftly, incorporating the role of financial incentives and penalties into the system. In addition to creating a game-engine (or similar) version of the NetLogo model, work is also underway to enable the system to learn from the consequences of its own behaviour, creating an optimisation algorithms through reinforcement learning or genetic algorithms capabilities that can ‘breed’ optimal policy combinations to drive the system from any current state to any desired future state.

In summary, though simple, the presented WorkSim model shows considerable potential in assisting workers’ compensation scheme managers better understand and explore the effect of policy and management scheme decisions on the performance of schemes ahead of time. Future iterations of this and associated models may prove valuable in ensuring the viability and health of schemes and the people they exist to serve, alike.

## Supplementary Information

Below is the link to the electronic supplementary material.

Supplementary file 1 (DOCX 5554 kb)
