# Supplementary material for: Development of a Computational Policy Model for Comparing the Effect of Compensation Scheme Policies on Recovery After Workplace Injury
Source: J Occup Rehabil. 2022 May 10;32(2):241–51. doi: 10.1007/s10926-022-10035-w (PMC9087158; doi:10.1007/s10926-022-10035-w)
Supplement: Supplementary file 1 — (DOCX 5554 kb) [file 10926_2022_10035_MOESM1_ESM.docx]

**Appendix A**


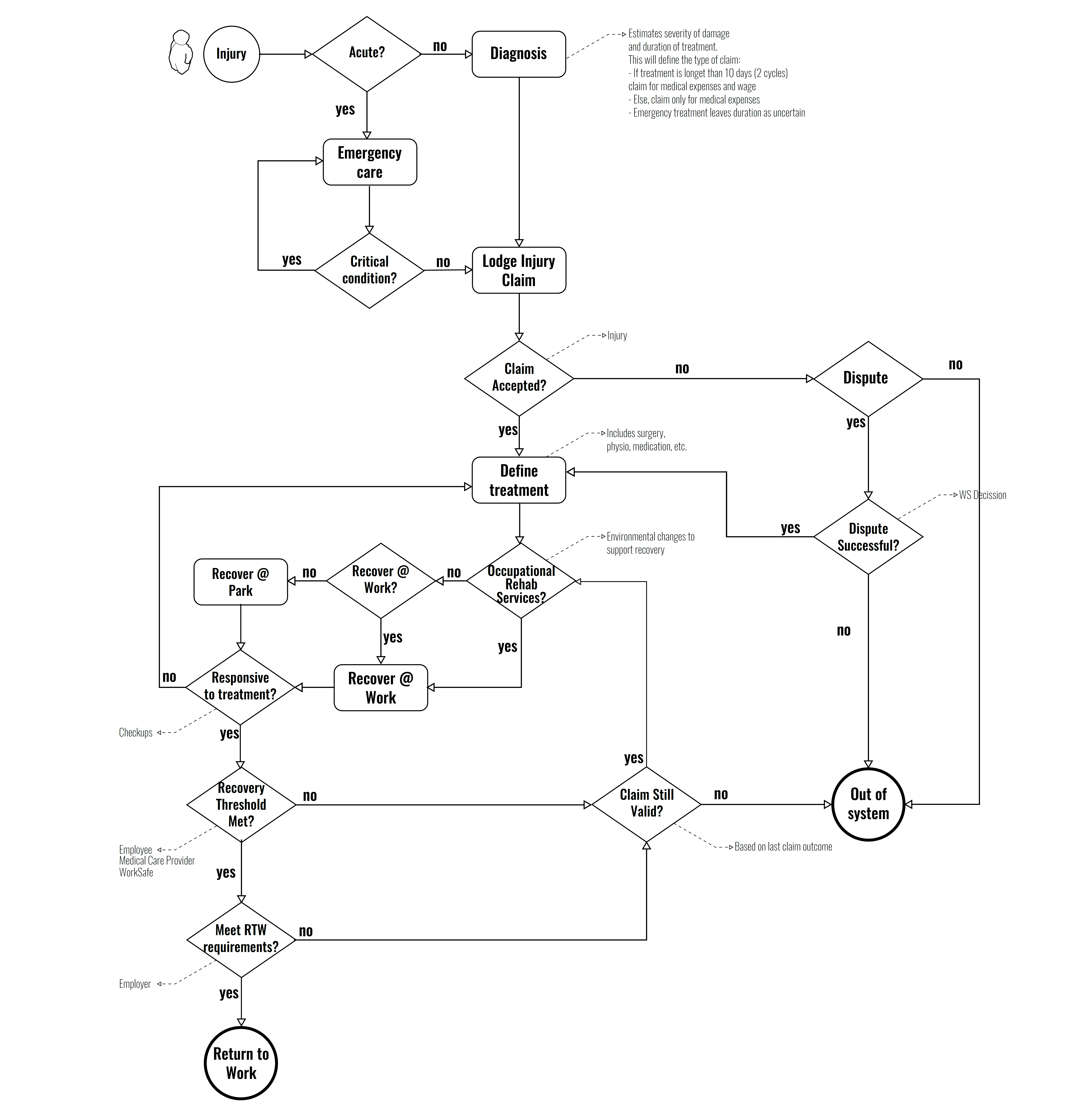


**Appendix B**


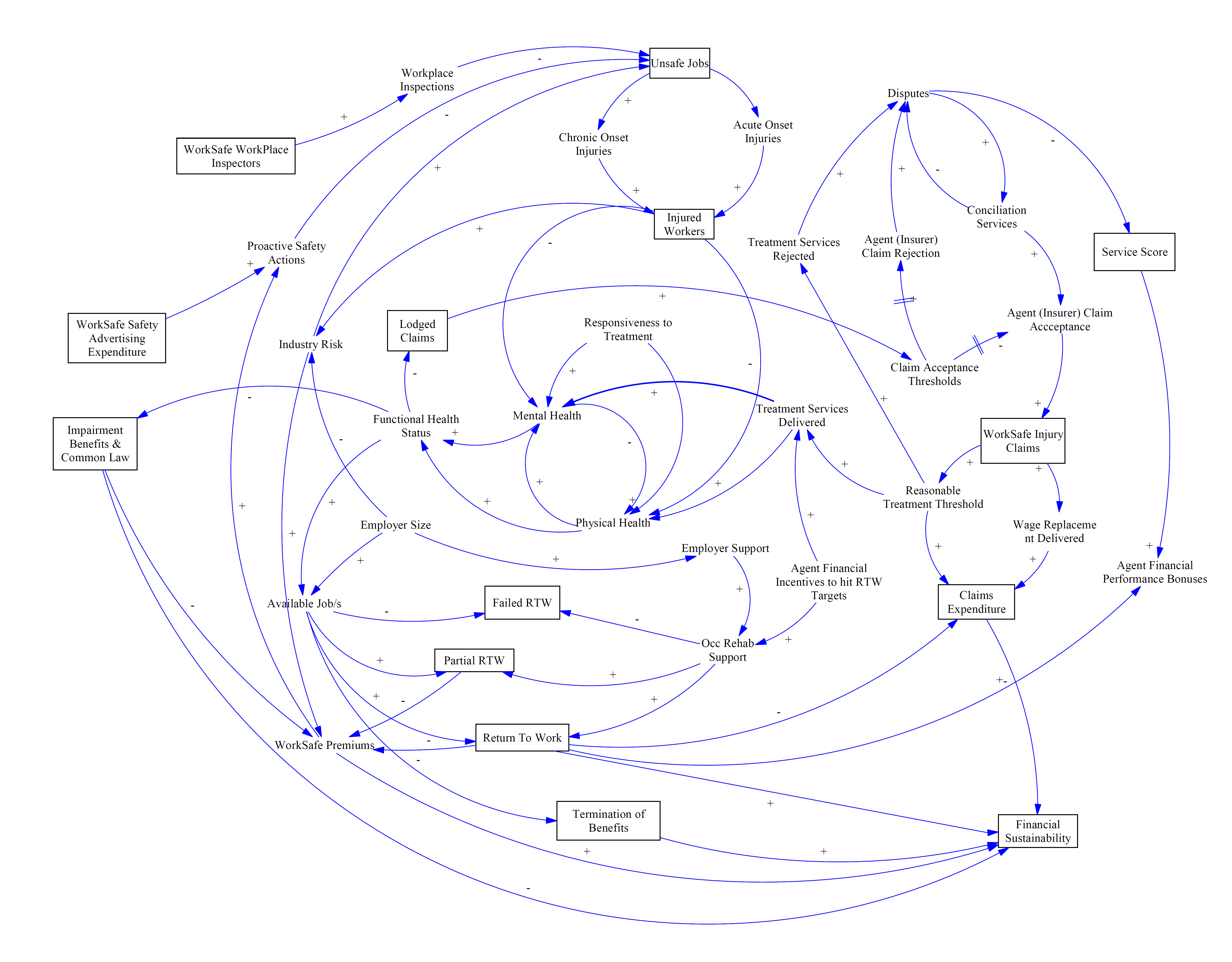


**Appendix C.**


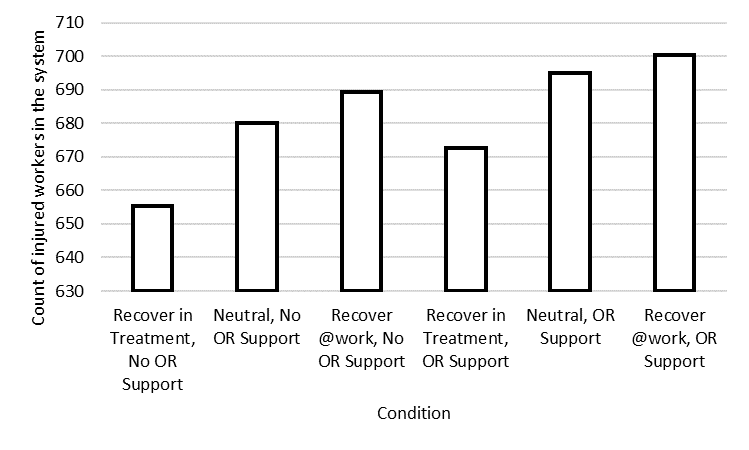


Figure 1. Mean count of workers in the system under each of 6 conditions.

Figure 2. Mean claim duration of workers in the system under each of 6 conditions.

Figure 3. Mean count of workers in the RTW pool under each of 6 conditions.


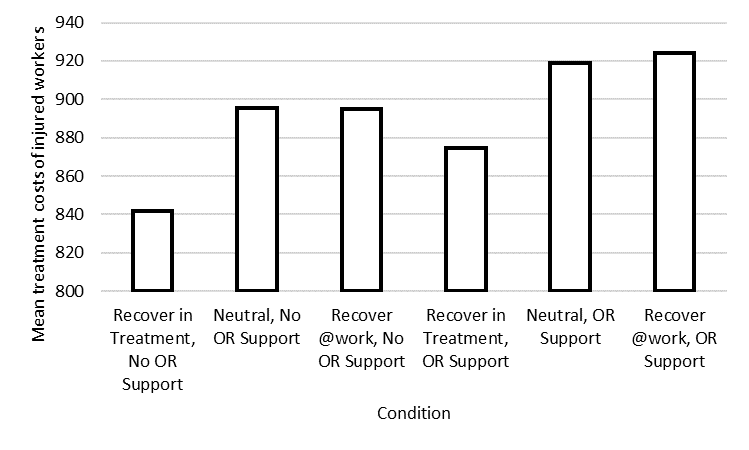


Figure 4. Mean treatment costs of workers in the system under each of 6 conditions.


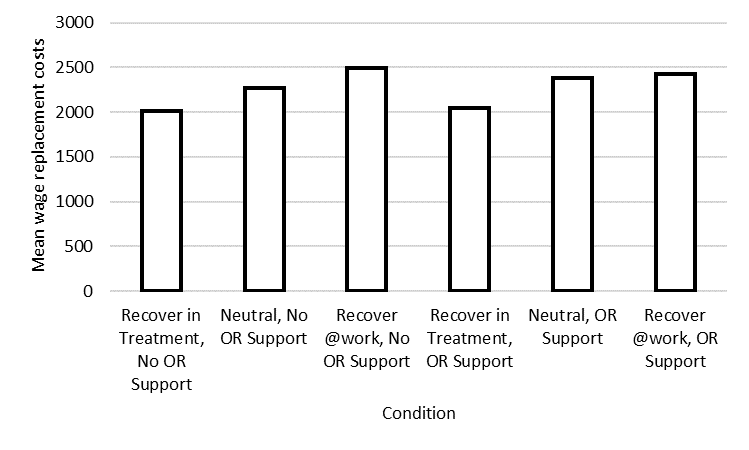


Figure 5. Mean wage replacement costs of workers in the system under each of 6 conditions.

Figure 6. Mean total system costs under each of 6 conditions.


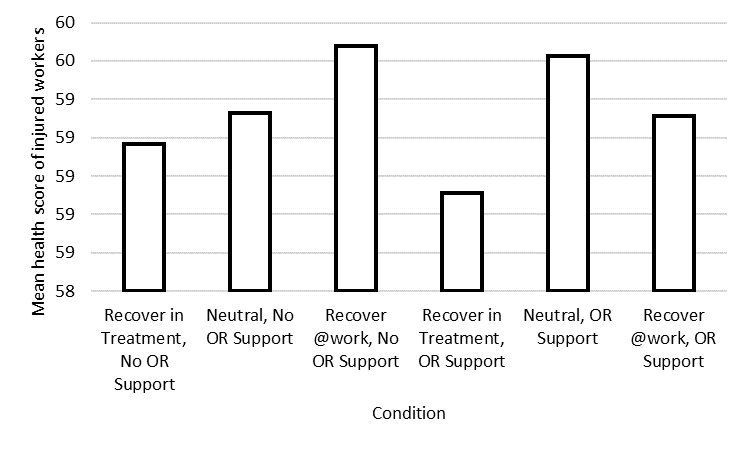


Figure 7. Mean health of Injured Workers under each of 6 conditions.

Figure 8. Mean satisfaction with the system of Injured Workers under each of 6 conditions.


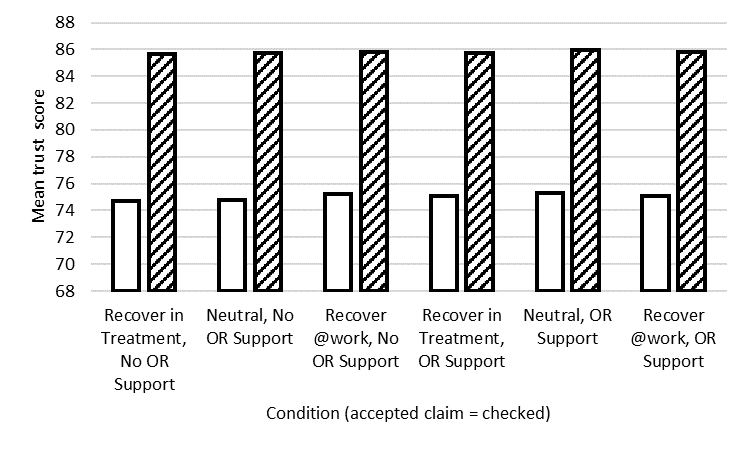


Figure 9. Mean trust that Injured Workers have in the system whether their claim has been accepted (checked) or rejected (blank) under each of 6 conditions.

**Appendix D**

globals [ NoRecoverycount

DisputeCount TotalSystemCosts]

breed [ VicPops VicPop ]

breed [ AcuteCares AcuteCare ]

breed [ GPs GP ]

breed [ ClaimAccepteds ClaimAccepted ]

breed [ NoRecoverys NoRecovery ]

breed [ TreatmentCentres TreatmentCentre ]

breed [ Disputes Dispute ]

breed [ Employer1s Employer1 ]

breed [ RTWs RTW ]

breed [ OccRehabProviders OccRehabProvider ]

breed [ LodgeClaims LodgeClaim ]

breed [ Workers Worker ]

breed [ OccRehabResources OccRehabResource ]

breed [ Advertisements Advertisement ]

OccRehabResources-own [ Addcap costofAddcap ]

Workers-own ; Attributes that individual Workers have or are in at any stage

[

State1

InGP

InEmergency

InNoRecovery

InClaimAccepted

InTreatment

InDispute

InOccRehabProvider

InEmployer1

InRTW

InLodgeClaim

PartialRTW

FullRTW

FailedRTW

GoingtoGP

GoingtoAcuteCare

GoingtoNoRecovery

GoingtoClaimAccepted

GoingtoTreatment

GoingtoDispute

GoingtoOccRehabProvider

GoingtoEmployer1

GoingtoRTW

GoingtoLodgeClaim

GoingtoVicPops

DynClaimTime

FinalClaimTime

InSystem ;; They have had their claim accepted

Trust ;; Trust in the system that increases if they have a good experience and decreases if their expectations aren't met

Memory ;; A boolean memory variable related to their recollection of past treatment or service events

Memory_Span ;; They forget abut their previous experiences after a certain time-span

Health ;; They have an incoming health variable related to their injury / 100

ClaimType ;; A boolean flag determining if their injury is a mental health claim

Satisfaction ;; Overall satisfaction score / 100 rises with treatment and access, decreases with disputes - need to link it to health

Entrytime ;; The tick that the worker's claim is accepted by the injury rehabilitation system

Timenow ;; Ticks on initial referral to Scheme Claim

Timenow1 ;; Tiicks related to ultimate assessment of eligibility

saliencyexpectation ;; How connected the expectation of service is to the experience

saliencyexperience ;; How connected the experience is to the expectation

initialassociationstrength ;; The association (essentially learning rate ) between the worker's experience of the system and how they react to it.

newv ;; used in calculatio nof the above

newassociationstrength ;; as above

vmax ;; maximum association

vmin ;; minimum association

speed ;; speed of the agent through the network across the plane

LodgeClaimExpectations ;; expectations of how long it will take for their claim to be accepted

engaged ;; Flag for if the worker remembers events

Responsiveness ;; How responsive the worker is to treatment provided

CostsTreatment ;; Costs of treatment received

CostsWageReplacement ;; Costs of Wage Replacement

Salary ;; Salary of the individual

;; need to add costs

]

Employer1s-own [

Readiness ;; How ready the employer is to take the worker back with adjusted duties

]

to setup

clear-all

ask patches [ set pcolor black ]

create-GPs 1 [ set shape "GP" set size 5 set label "GP" set xcor 13.92 set ycor 42.25 set color brown]

create-AcuteCares 1 [ set shape "ambulance" set size 5 set label "Emergency Care" set xcor 6.35 set ycor 33.52 set color red ]

create-LodgeClaims 1 [ set shape "Insurer" set size 5 set label "LodgeClaim" set xcor 4.71 set ycor 22.1 set color white set heading 0 ]

create-ClaimAccepteds 1 [ set shape "computer workstation" set size 5 set label "Accepted_Claim" set xcor 9.51 set ycor 11.58 set color white ]

create-NoRecoverys 1 [ set shape "Garbage Can" set size 5 set label "No Recovery" set xcor 19.22 set ycor 5.33 set color white]

create-TreatmentCentres 1 [ set shape "Health Care" set size 5 set label "Treatment Centre" set xcor 30.77 set ycor 5.33 set color white ]

create-Disputes 1 [ set shape "Face sad" set size 5 set label "Disputes" set xcor 40.49 set ycor 11.58 set color red ]

create-Employer1s 1 [ set shape "Building" set size 5 set label "Employer" set xcor 45.29 set ycor 22.08 set color white set readiness random-normal 1 .1 ]

create-RTWs 1 [ set shape "pool" set size 5 set label "Return to Work Pool" set xcor 36.08 set ycor 42.25 set color red ]

create-OccRehabProviders 1 [ set shape "OR" set size 5 set label "Occ Rehab Provider" set xcor 43.65 set ycor 33.52 set color yellow ]

create-VicPops 1 [ set shape "Factory" set xcor 25 set ycor 25 set size 5 set label "General Population" set xcor 25 set ycor 45.5 set color white ]

create-OccRehabResources 1 [ set shape "OR1" set color blue move-to one-of OccRehabProviders set Addcap 1 set CostofAddCap AddCap ]

ask turtles [ create-links-with other turtles ]

create-Advertisements 1 [ set shape "Advert" set xcor 25 set ycor 25 Set size 5 ]

ask links [set color white ]

create-workers 10 [ set shape one-of [ "Worker1" "Worker2"] set state1 0 move-to one-of VicPops set trust random-normal 80 3 set speed random-normal 1 .1 set size 2]

ask workers [ set satisfaction random-normal 70 5 set responsiveness random-normal 1 .01 resettrust set memory_Span random-normal Memoryspan 30 set memory 0 set initialassociationstrength InitialV

set saliencyExpectation random-normal ExpectationSaliency .1 set SaliencyExperience random-normal ExperienceSaliency .1 set LodgeClaimExpectations ManageExpectations

set health random-normal 50 10 isClaimType set salary random-normal 55 10 set salary (salary ^ 1.2) ]

setup-image

set Injured_workers 10

reset-ticks

end

to isClaimType

set ClaimType one-of [ 0 1 2 ]

if claimType = 0 [ set label "A" ]

if claimType = 1 [ set label "C" ]

if claimType = 2 [ set label "M" ]

end

to setup-image

import-drawing "wslogo.png"

set Adspend 5

set SendORs false

end

to resettrust

if trust > 100 or trust < 1 [ set trust random-normal 80 3 ]

if saliencyExpectation > 1 or saliencyExpectation <= 0 [ set saliencyExpectation random-normal ExpectationSaliency 10 ]

if saliencyExperience > 1 or saliencyExperience <= 0 [ set saliencyExpectation random-normal ExpectationSaliency 10 ]

if health > 100 or health < 1 [ set health random-normal 50 10 ]

if satisfaction > 100 or satisfaction < 10 [ set satisfaction trust ]

if salary < 25 [ set salary random-normal 55 20 ]

end

to go

ask workers [

Initialise

GPreferral

Emergency

Disputesincare

DisputetoGeneral

TestDispute

BecomeAcceptedClaim

AccessTreatment

TreatmenttoEmployer

DisputeToClaimAccepted

ReturntoWork

EmployernotReady

BecomeLodgeClaim

EmployerfromGP

ClaimAfterMisDiagnosis

TreatmentToGeneral

Captrust

CalculateTrustFactor

Rememberevents

Resetinitial

LimitInitialAssociation

OverCapReview

OverCapNew

SocialEpi

EngageExpectations

RemoveHealthyWorkers

CountTreatmentCosts

CountWageReplacementCosts

ChangeHealth

TimeOut

Changeshapeworkers

]

ask OccRehabResources [ GoHelp ChangeAddcap changeshape ]

ask Advertisements [ changecolor ]

ask OccRehabResources [ changecolor ]

ask Employer1s [ Recalculatereadiness ]

ask turtles [

set size (5 + sqrt count Workers in-radius 1 )

]

ask Workers [ set size 2]

ask Advertisements [ set size AdSpend ]

createClaimAccepteds

countNoRecoverys

;; burnpatches

EstimateTotalSystemCosts

;; if ticks = 2000 [ stop ]

tick

end

to capTrust

if trust > 100 [ set trust Maxtrust ]

if trust < 1 [ set trust MinTrust ]

end

to initialise

set state1 1

end

to gpreferral ;; individuals emerging from the general population into GPs

if GP_referral > random 100 and state1 = 1 and any? VicPops-here [

face one-of GPs fd speed set goingtoGP 1 set state1 0 ]

if goingtoGP = 1 [ face one-of GPs fd speed ]

if any? GPs in-radius 1 [ move-to one-of GPs set InGP 1 set goingtoGP 0 set state1 0 set CostsTreatment Coststreatment + .1 ]

end

to Emergency ;; individuals emerging from the general population into emergency areas of hospitals

if Emergency_Pres > random 100 and state1 = 1 and InEmergency = 0 and any? VicPops-here [

face one-of AcuteCares fd speed set GoingtoAcuteCare 1 set State1 0 ]

if GoingtoAcuteCare = 1 [ face one-of AcuteCares fd speed ]

if any? AcuteCares in-radius 1 [ move-to one-of AcuteCares set InEmergency 1 set InGP 0 set GoingtoAcuteCare 0 set CostsTreatment Coststreatment + 1 ]

end

to BecomeLodgeClaim ;;

if Emergency_Referral > random 100 and InEmergency = 1 and any? AcuteCares-here and health < Claim_Threshold [

face one-of LodgeClaims fd speed set goingtoLodgeClaim 1 set InEmergency 0 ]

if goingtoLodgeClaim = 1 [ Face one-of LodgeClaims fd speed ]

if any? LodgeClaims in-radius 1 [ move-to one-of LodgeClaims Set InLodgeClaim 1 set InEmergency 0 set GoingtoLodgeClaim 0 ]

if GP_Referral > random 100 and InGP = 1 and any? GPs-here and health < Claim_Threshold [

face one-of LodgeClaims fd speed set goingtoLodgeClaim 1 set InGP 0 ]

if goingtoLodgeClaim = 1 [ Face one-of LodgeClaims fd speed ]

if any? LodgeClaims in-radius 1 [ move-to one-of LodgeClaims Set InLodgeClaim 1 set InGP 0 set GoingtoLodgeClaim 0 ]

end

to BecomeAcceptedClaim

if Label = "A" and InLodgeClaim = 1 and any? LodgeClaims-here

and count Workers with [ InClaimAccepted = ( 1 * OverbookingRate )] < Assessment_Capacity and (random-normal 0.9 .1 ) * (random-normal 0.8 .1 ) > Accept_Threshold [

face one-of ClaimAccepteds fd speed set goingtoClaimAccepted 1 Set InLodgeClaim 0 ]

if Label = "C" and InLodgeClaim = 1 and any? LodgeClaims-here

and count Workers with [ InClaimAccepted = ( 1 * OverbookingRate )] < Assessment_Capacity and (random-normal 0.6 .1 ) * (random-normal 0.8 .1 ) > Accept_Threshold [

face one-of ClaimAccepteds fd speed set goingtoClaimAccepted 1 Set InLodgeClaim 0 ]

if Label = "M" and InLodgeClaim = 1 and any? LodgeClaims-here

and count Workers with [ InClaimAccepted = ( 1 * OverbookingRate )] < Assessment_Capacity and (random-normal 0.4 .1 ) * (random-normal 0.8 .1 ) > Accept_Threshold [

face one-of ClaimAccepteds fd speed set goingtoClaimAccepted 1 Set InLodgeClaim 0 ]

if any? ClaimAccepteds in-radius 1 [ move-to one-of ClaimAccepteds Set InClaimAccepted 1 Set InLodgeClaim 0 set goingtoClaimAccepted 0 set InSystem 1 set Entrytime ticks ]

end

to AccessTreatment

if InClaimAccepted = 1 and any? ClaimAccepteds-here and count Workers with [ InTreatment = 1 ] < Treatment_Capacity [

face one-of TreatmentCentres fd speed set Goingtotreatment 1 Set InClaimAccepted 0 ]

if GoingtoTreatment = 1 [ face one-of TreatmentCentres fd speed ]

if any? TreatmentCentres in-radius 1 [ move-to one-of TreatmentCentres Set InTreatment 1 Set InClaimAccepted 0 set GoingtoTreatment 0 set health (health + ((100 - health) * .05 ) * Responsiveness )]

end

to OverCapNew

if inClaimAccepted = 1 and count Workers with [ inClaimAccepted = 1 ] > Assessment_Capacity [

face one-of LodgeClaims fd speed set goingtoLodgeClaim 1 set InClaimAccepted 0 ]

if goingtoLodgeClaim = 1 [ Face one-of LodgeClaims fd speed ]

if any? LodgeClaims in-radius 1 [ move-to one-of LodgeClaims Set InLodgeClaim 1 set InClaimAccepted 0 set GoingtoLodgeClaim 0 ]

end

to OverCapReview

if InTreatment = 1 and count Workers with [ InTreatment = 1 ] > Treatment_Capacity [

face one-of ClaimAccepteds fd speed set goingtoClaimAccepted 1 set InTreatment 0 ]

if goingtoClaimAccepted = 1 [ Face one-of ClaimAccepteds fd speed ]

if any? ClaimAccepteds in-radius 1 [ move-to one-of ClaimAccepteds Set InClaimAccepted 1 set InTreatment 0 set goingtoClaimAccepted 0 ]

end

to TreatmentToGeneral

if health > Claim_Threshold and InTreatment = 1 and any? TreatmentCentres-here [ ;; people are more likely resist going back to work if their levels of trust are lower

face one-of RTWs fd speed set GoingtoRTW 1 set InTreatment 0 ]

if GoingtoRTW = 1 [ face one-of RTWs fd speed ]

if any? RTWs in-radius 1 [ move-to one-of RTWs set InRTW 1 set GoingtoRTW 0 set FinalClaimTime DynClaimTime ]

end

to TreatmenttoEmployer ;; in here is where trust is going to affect the DNA rate

if (health + (PromoteRecoveryAtWork + random 5 - random 5 )) > Claim_Threshold and InTreatment = 1 and any? TreatmentCentres-here [ ;; people are more likely resist going back to work if their levels of trust are lower

face one-of Employer1s fd speed set GoingtoEmployer1 1 set InTreatment 0 ]

if GoingtoEmployer1 = 1 [ face one-of Employer1s fd speed ]

if any? Employer1s in-radius 1 [ move-to one-of Employer1s Set InEmployer1 1 set InTreatment 0 set GoingtoEmployer1 0 ]

end

to EmployerFromGP ;; trust is going affect the DNA rate here

if any? GPs-here and health > (Claim_Threshold - DiagnosisError ) [

face one-of Employer1s fd speed set GoingtoEmployer1 1 set InGP 0 ]

if GoingtoEmployer1 = 1 [ face one-of Employer1s fd speed ]

if any? Employer1s in-radius 1 [ move-to one-of Employer1s Set InEmployer1 1 set InGP 0 set GoingtoEmployer1 0 ]

end

to TestDispute

if InSystem = 0 and Success_Dispute_% > random 100 and InDispute = 1 and any? Disputes-here and ((100 - trust ) > 1000 )[ ;; so this send people who have a successful claim dispute back to the Claim Lodgement stage - they are not in the system yet

face one-of LodgeClaims fd speed set GoingtoLodgeClaim 1 set InDispute 0 set trust (trust * .9) ]

if GoingtoLodgeClaim = 1 [ face one-of LodgeClaims fd speed set indispute 0 ]

if any? LodgeClaims in-radius 1 [ move-to one-of LodgeClaims Set InLodgeClaim 1 set GoingtoLodgeClaim 0 ]

end

to DisputetoGeneral

if InSystem = 0 and Success_Dispute_% < random 100 and InDispute = 1 and any? Disputes-here [ ;; so this send people who have an unsuccessful claim dispute back to the general population of workers

face one-of VicPops fd speed set GoingtoVicPops 1 set InDispute 0 set size 10 ]

if GoingtoVicPops = 1 [ face one-of VicPops fd speed set InDispute 0 ]

if any? VicPops in-radius 1 [ move-to one-of VicPops die ]

end

to DisputeToClaimAccepted

if InSystem = 1 and Success_Dispute_% > random 100 and InDispute = 1 and any? Disputes-here [

face one-of ClaimAccepteds fd speed set GoingToClaimAccepted 1 set trust (trust * .9) set satisfaction satisfaction * .9 set CostsTreatment (CostsTreatment + one-of [ -1 0 ]) ]

if GoingToClaimAccepted = 1 [ face one-of ClaimAccepteds fd speed if any? ClaimAccepteds in-radius 1 [ move-to one-of ClaimAccepteds set InClaimAccepted 1 set GoingToClaimAccepted 0 set health (health * responsiveness) set indispute 0 ]]

end

to EmployernotReady ;; trust is going to affect the likelihood that anyone comes ouut of DNA1 back to review here

if [ readiness ] of one-of Employer1s > 1 and any? Employer1s-here [

face one-of RTWs fd speed set GoingtoRTW 1 Set InRTW 0 set FullRTW 1 ]

if GoingtoRTW = 1 [ face one-of RTWs fd speed ]

if any? RTWs in-radius 1 [ move-to one-of RTWs Set InRTW 1 Set InEmployer1 0 set GoingtoRTW 0 set fullRTW 1 set FinalClaimTime DynClaimTime ]

;; trust is going to affect the likelihood that anyone comes ouut of DNA1 back to review here

if Trust > random 100 and InRTW = 1 and any? RTWs-here [

face one-of VicPops fd speed set GoingtoVicPops 1 Set InRTW 0 set FullRTW 1 ]

if GoingtoVicPops = 1 [ face one-of VicPops fd speed ]

if any? VicPops in-radius 1 [ move-to one-of VicPops Set InRTW 0 die ]

end

to ReturntoWork ;;

if any? OccRehabResources-here and ( health * ([ Readiness ] of one-of Employer1s ) * ([ AddCap ] of one-of OccRehabResources)) > Claim_Threshold and InEmployer1 = 1 and

any? Employer1s-here [

face one-of RTWs fd speed set GoingtoRTW 1 set InEmployer1 0 set Coststreatment (CostsTreatment + OccRehabMultiplier ) set PartialRTW 1 ]

if not any? OccRehabResources-here and ( health * ([ Readiness ] of one-of Employer1s ) ) < Claim_Threshold and InEmployer1 = 1 and

any? Employer1s-here and Insystem = 1 [

face one-of TreatmentCentres fd speed set GoingtoTreatment 1 set InEmployer1 0 set salary (salary * ( health / Claim_Threshold )) set FailedRTW 1 set PartialRTW 0 set FullRTW 0 ]

if GoingtoTreatment = 1 [ face one-of TreatmentCentres fd speed if any? TreatmentCentres in-radius 1 [ move-to one-of TreatmentCentres Set InTreatment 1 set InEmployer1 0 set GoingtoTreatment 0 ]]

end

to ReturntoWorkwithOccRehab ;; trust is going to affec the DNA2 rate here

if GoingtoVicPops = 1 [ face one-of VicPops fd speed if any? VicPops in-radius 1 [ move-to one-of VicPops die ]]

if (health * ([ Readiness ] of one-of Employer1s ) ) > Claim_Threshold and InRTW = 1 and any? RTWs-here [

face one-of VicPops fd speed set GoingtoVicPops 1 set InRTW 0 set FinalClaimTime DynClaimTime ]

if GoingtoVicPops = 1 [ face one-of VicPops fd speed if any? VicPops in-radius 1 [ move-to one-of VicPops die ]] ;; then people need to actually ret from the pool

end

to ClaimAfterMisdiagnosis

if InSystem = 0 and any? Employer1s-here and health < Claim_Threshold and inEmployer1 = 1 [

face one-of LodgeClaims fd speed set GoingtoEmployer1 0 set inEmployer1 0 set goingtoLodgeClaim 1 ]

if GoingtoLodgeClaim = 1 [ face one-of LodgeClaims fd speed ]

if any? LodgeClaims in-radius 1 [ move-to one-of LodgeClaims Set InLodgeClaim 1 set GoingtoLodgeClaim 0 ]

end

to Disputesincare

if ((100 - trust ) / 10 ) > random 100 and InLodgeClaim = 1 and any? LodgeClaims-here [

face one-of Disputes fd speed set GoingtoDispute 1 set color red ]

if GoingtoDispute = 1 [ face one-of Disputes fd speed if any? Disputes in-radius 1 [ move-to one-of Disputes set GoingtoDispute 0 set InDispute 1 set InLodgeClaim 0 set satisfaction satisfaction * .9 set trust trust * .5 ]]

if ((100 - trust ) / 10 ) > random 100 and InTreatment = 1 and any? TreatmentCentres-here [

face one-of Disputes fd speed set GoingtoDispute 1 set color red ]

if GoingtoDispute = 1 [ face one-of Disputes fd speed if any? Disputes in-radius 1 [ move-to one-of Disputes set GoingtoDispute 0 set InDispute 1 set InTreatment 0 set satisfaction satisfaction * .9 set trust trust * .5 ]]

end

to CountNoRecoverys

set NoRecoverycount ( count Workers with [ goingtoNoRecovery = 1 ] )

set DisputeCount (( count Workers with [ GoingtoDispute = 1 ]) + (count workers with [ InDispute = 1 ] ) )

end

;;need to combine Occrehab and Employer Readiness

to EngageExpectations

if goingtoLodgeClaim = 1 [ set timenow1 ticks ] ;; need this to record once and then forget about it

if any? LodgeClaims-here and ticks - timenow1 > (LodgeClaimexpectations + random Error_of_Estimate - random Error_of_Estimate) [ rememberevents set engaged true ] ;; OK, so now timmenow only starts at the point at which people go into the LodgeClaim

end

to rememberevents

if any? LodgeClaims-here and engaged = true [ set memory 1 set timenow ticks ]

;; add in more conditions here

if any? TreatmentCentres-here [ set memory 1 set timenow ticks ]

if any? ClaimAccepteds-here [ set memory 1 set timenow ticks ]

if any? acuteCares-here [ set memory 1 set timenow ticks ]

if any? GPs-here and memory = 1 [ set timenow ticks ]

if ticks - timenow > memoryspan [ set memory 0 set trust trust ] ;; it needs to do nothing if memory = 0 here. Trust needs to go up if a good thing happens, that's all.

end

to calculatetrustfactor

if memory = 1 and any? LodgeClaims-here [ set newv ( ( saliencyExpectation * SaliencyExperience ) * (( (MaxTrust / 100) - initialassociationstrength ) ))

set newassociationstrength ( initialassociationstrength + newv ) set trust trust - newassociationstrength ]

;;add in more here

if memory = 1 and any? TreatmentCentres-here [ set newv ( ( saliencyExpectation * SaliencyExperience ) * (( (MaxTrust / 100) - initialassociationstrength ) ))

set newAssociationStrength ( initialassociationstrength - newv ) set trust trust + newassociationstrength]

if memory = 1 and any? ClaimAccepteds-here [ set newv ( ( saliencyExpectation * SaliencyExperience ) * (( (MaxTrust / 100) - initialassociationstrength ) ))

set newassociationstrength ( initialassociationstrength - newv ) set trust trust + newassociationstrength]

if memory = 1 and any? acuteCares-here [ set newv ( ( saliencyExpectation * SaliencyExperience ) * (( (MaxTrust / 100) - initialassociationstrength ) ))

set newassociationstrength ( initialassociationstrength - newv ) set trust trust + newassociationstrength]

set vmax (MaxTrust / 100) set vmin MinTrust

if newv > (MaxTrust / 100) [ set newv (MaxTrust / 100) ]

if newv < MinTrust [ set newv MinTrust ]

if saliencyExpectation > 1 [ set saliencyExpectation 1 ]

if saliencyExpectation <= 0 [ set saliencyExpectation 0 ]

if saliencyExperience > 1 [ set SaliencyExperience 1 ]

if saliencyExperience <= 0 [ set SaliencyExperience 0 ]

end

to resetinitial

if newassociationstrength <= MaxTrust [ set initialassociationstrength ( newassociationstrength ) ]

end

to createClaimAccepteds

create-Workers (Injured_Workers * (1 - (AdSpend / 100))) [ set shape one-of [ "Worker1" "Worker2" ] set size 2 set state1 1 move-to one-of VicPops set speed random-normal 1 .1

set trust random-normal 80 3 set satisfaction random-normal 70 5 set responsiveness random-normal 1 .01 set memory_Span random-normal Memoryspan 30 set memory 0 set initialassociationstrength InitialV

set saliencyExpectation random-normal ExpectationSaliency .1 set SaliencyExperience random-normal ExperienceSaliency .1 set LodgeClaimExpectations ManageExpectations set health random-normal 50 10 IsClaimType

set salary random-normal 55 10 set salary (salary ^ 1.2) resettrust

] ;;ifelse any? Workers with [ GoingtoVicPops = 1 ] and Expectation > random 100 set trust mean [ trust ] of Workers with [ GoingtoVicPops = 1 ] ][ set trust random-normal 80 10 resettrust

end

to limitInitialAssociation

if initialassociationstrength < InitialV [ set initialassociationstrength InitialV ]

end

to SocialEpi

if any? other Workers-here with [ trust < [ trust ] of myself ] [ set trust trust - 1 ]

if any? other Workers-here with [ trust > [ trust ] of myself ] [ set trust trust + 1 ]

end

to RemoveHealthyWorkers

if InSystem = 0 and health > Claim_Threshold [ die ]

end

to CountTreatmentCosts

if InSystem = 1 and any? TreatmentCentres-here [ set CostsTreatment CostsTreatment + random-normal .1 .02 ]

end

to CountWageReplacementCosts

if InSystem = 1 [ set CostsWageReplacement CostsWageReplacement + (Salary / 365 * .8 ) ]

end

to EstimateTotalSystemCosts

set TotalSystemCosts (sum [ CostsWageReplacement ] of workers + sum [ CostsTreatment ] of workers + AdSpend * 10 )

end

to timeout

if any? Norecoverys-here [ die ]

if InSystem = 1 and ticks - entrytime > max_claim_duration [ set size 20 set color yellow face one-of NoRecoverys set InSystem 2 fd speed set state1 0 set goingtoAcutecare 0 set InEmergency 0 set GoingtoTreatment 0 set Intreatment 0

set GoingtoClaimAccepted 0 set inClaimAccepted 0 set GoingtoRTW 0 set inRTW 0 set GoingtoVicPops 0 set goingtoEmployer1 0 set inEmployer1 0 set goingtoGP 0 set GoingtoLodgeClaim 0 set GoingtoAcuteCare 0 ]

if InSystem = 2 [ face one-of NoRecoverys set InSystem 2 fd speed if any? NoRecoverys in-radius 1 [ move-to one-of NoRecoverys ] ]

set dynclaimtime ( ticks - entrytime )

end

to GoHelp

if SendORs = true [ face one-of Employer1s fd .5 if any? Employer1s in-radius 1 [ move-to one-of Employer1s ]]

if SendORs = false [ face one-of OccRehabProviders fd .5 if any? OccRehabProviders in-radius 1 [ move-to one-of OccrehabProviders ] ]

end

to ChangeHealth

set health health + one-of [ -1 0 1 ]

end

to ChangeAddcap

set AddCap random-normal ORCapacity .1

end

To Recalculatereadiness

set readiness random-normal (1 + (Adspend / 300 )) .1

end

to changeshape

ifelse remainder ticks 10 < 5 [ set shape "OR1" ] [ set shape "OR2" ]

end

to changeshapeworkers

ifelse remainder dynclaimtime 10 < 5 [ set shape "OR1" ] [ set shape "OR2" ]

end

to changecolor

set color (white + random 2 - random 2)

end
